# A decade after the Fundão Dam collapse: key findings from studies in the Doce River basin and adjacent coastal and marine environments

**DOI:** 10.1007/s10661-026-15356-4

**Published:** 2026-05-07

**Authors:** Fabio Cavalca Bom, Fabiana de Matos Costa, Gisele Daiane Pinha, Manuela Santos Santana, Nelson de Almeida Gouveia, Beatrice Padovani Ferreira, Maikon Di Domenico, Nadson Ressye Simões, Alex Cardoso Bastos, Fabian Sá, Kyssyanne Samihra Santos Oliveira

**Affiliations:** 1 Laboratório de Geoquímica Ambiental e Poluição Marinha (LabGAm), Departamento de Oceanografia e Ecologia, Universidade Federal do Espírito Santo, Vitória, Espírito Santo, Brazil; 2 Laboratório de Estudos e Conservação de Sistemas Aquáticos (LECSA), Universidade Federal do Sul da Bahia, Itabuna, Bahia Brazil; 3 Laboratório de Ecologia Marinho (ECOMar), Universidade Federal Do Paraná, Pontal Do Paraná, Paraná, Brazil; 4 Laboratório de Estudos em Ecossistemas Oceânicos e Recifais (LECOR), Departamento de Oceanografia, Universidade Federal de Pernambuco, Cidade Universitária, Av. Arquitetura S/N, Recife, PE Brazil; 5 Laboratório de Geociências Marinhas (LABOGEO), Departamento de Oceanografia e Ecologia, Universidade Federal do Espírito Santo, Vitória, Espírito Santo Brazil; 6 Laboratório de Estudos do Sistema Atmosfera-Continente-Oceano (ESOLab), Departamento de Oceanografia e Ecologia, Universidade Federal do Espírito Santo, Vitória, Espírito Santo Brazil; 7 University of Victoria, Victoria, BC Canada

**Keywords:** Environmental disaster, Mining impacts, Long-term monitoring, Systematic review

## Abstract

The Fundão tailings dam marks its 10th year of disaster in 2025, underscoring the need for a critical assessment of the studies related to the impacts on aquatic ecosystems caused by the largest disaster in mining history. In this context, the present study aimed to review articles that, throughout the years, have investigated changes in the Doce River basin, as well as in coastal and marine areas affected by this collapse. We applied a systematic analysis of the published literature, identifying the main results in relation to the methodologies applied. Therefore, the studies were characterized according to their methodological approaches, spatiotemporal efforts, environmental compartments, and topics of study evaluated. In total, 182 articles published between 2016 and July 2025 were considered. Considerable heterogeneity was observed regarding spatial and temporal coverage, ranging from point‐in‐time assessments to large-scale analyses. Most of the reviewed studies revealed both acute and chronic effects on water and sediment quality across all monitored environments, which in turn resulted in significant impacts on biodiversity. Some studies investigated all environments in an integrated manner, which made possible a deeper understanding of the connections between these systems, showing the importance of this type of approach. Together, the studies revealed the challenge of learning from disasters, especially regarding environmental issues. Furthermore, they encouraged a critical perspective for long-term monitoring planning and the next steps needed to address aquatic biota recovery. This synthesis underscores the critical need for long-term, integrated monitoring to effectively guide the recovery of these interconnected ecosystems.

**Supplementary Information:**

The online version contains supplementary material available at 10.1007/s10661-026-15356-4.

## Introduction

Mining is one of the oldest and most fundamental activities in the history of human civilization, being responsible for providing the raw materials essential to the production of most materials used today (Candeias et al., 2018). Yet, despite its great importance, mining can be considered one of the main anthropogenic activities contributing to changes in the landscape, through the mobilization of large amounts of rock and soil (Firozjaei et al., 2021). These changes can lead to impacts such as air and water pollution, soil/sediment contamination by metals, land subsidence, and geological disasters (Zhong et al., 2024, 2025). Moreover, this activity is related to losses of ecosystem services, associated with reductions in terrestrial animal populations and declines in the diversity of riparian vegetation and aquatic communities (Boldy et al., 2021; Damseth et al., 2024; Martins-Oliveira et al., 2021).

In addition to this major anthropogenic driver, mining also exerts pressure through the disposal of waste, usually stored in tailings dams, which in some cases have failed, representing a threat to human life and downstream ecosystems. Studies indicate that more than 300 dam failures have occurred since the beginning of the twentieth century (Islam & Murakami, 2021; Lin et al., 2022), caused by different factors such as dam structure and foundation conditions, earthquakes, mine subsidence, structural inadequacies, improper or failed decanting, external erosion, infiltration and internal erosion, overtopping, and slope instability. Of these, only 54 incidents were considered responsible for the loss of 2976 human lives over the past hundred years (Islam & Murakami, 2021), in addition to causing severe ecological damage to impacted environments (Fernandes et al., 2016; Garcia et al., 2025).

Among the reported accidents, some stand out: (1) for the large volume of tailings released into the environment, such as Brazil/2019 (9 million m^3^ of iron waste), Philippines/2012 (13 million m^3^ of copper waste), Canada/2014 (23.6 million m^3^ of gold and copper waste), Philippines/1992 (32.2 million m^3^ of copper waste), and Brazil/2015 (43 million m^3^ of iron waste); and (2) for the long distances traveled by tailings in watercourses, such as Canada/1990 (168 km of uranium waste contamination), Bolivia/1996 (300 km of lead-zinc waste contamination), Mexico/2014 (420 km of copper waste contamination), and Brazil/2015, with pollutants spreading along 668 km of rivers and reaching the Atlantic Ocean (Carmo et al., 2017).

### Fundão dam collapse

**Fig. 1:**
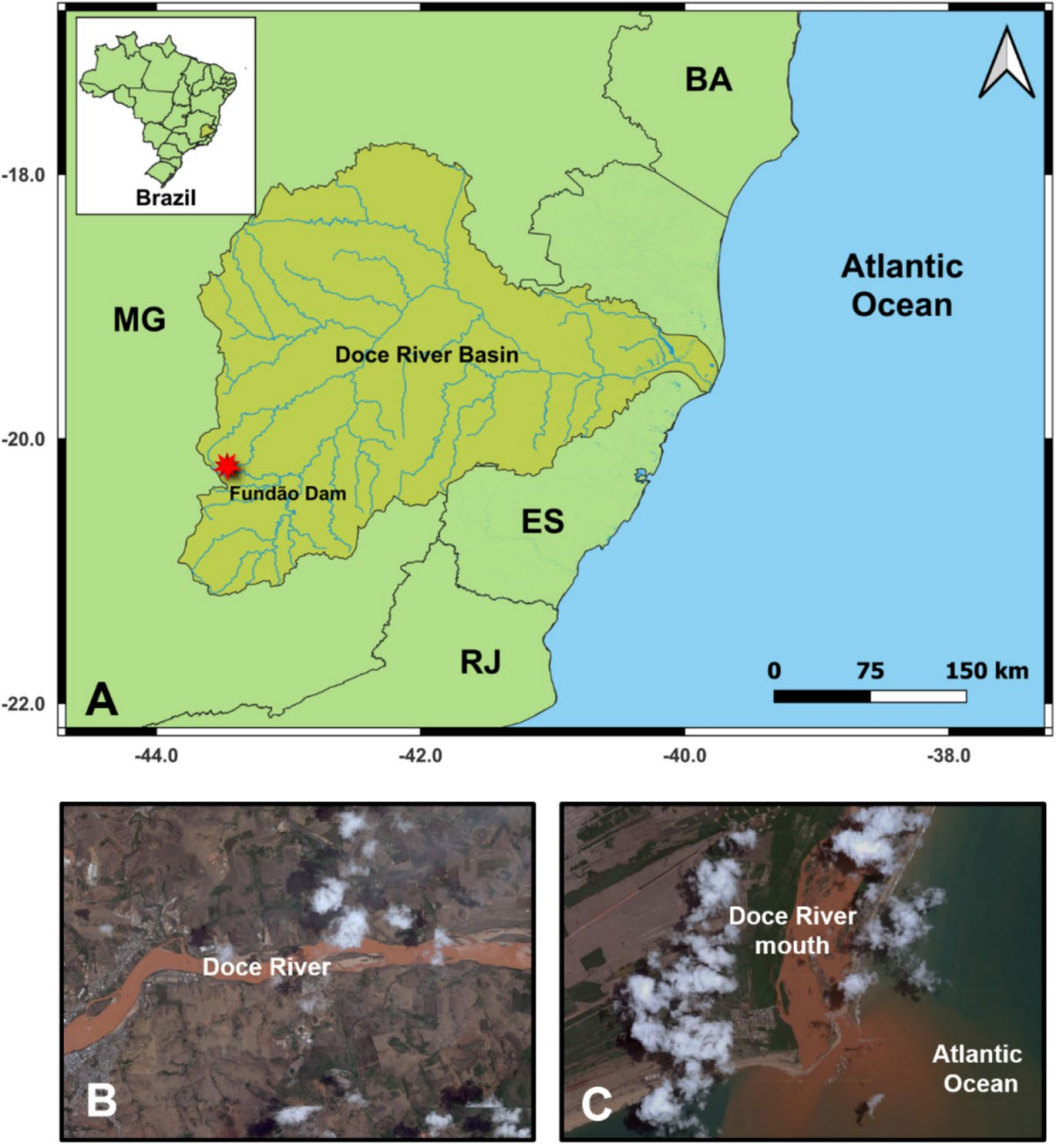
**A** Map of the Doce River basin and the adjacent coastal and marine regions. The location of the Fundão dam collapse is highlighted. **B** and **C** Satellite images from December 30, 2015, showing the presence of tailings along the course of the Doce River and in the adjacent coastal and marine region, respectively (Images: Google Earth)

One of the most environmentally significant of these events occurred in Brazil on November 5, 2015. The Fundão dam collapse, located in Mariana, Minas Gerais (Fig. 1), released tailings into the downstream valleys (Fonseca & Fonseca, 2016; Samarco, 2017) and is considered the biggest disaster in the history of mining worldwide. (Vaneli et al., 2022). The main cause of the Fundão tailings dam rupture was the liquefaction process, a geotechnical phenomenon in which saturated soils (filled with water) temporarily lose their resistance and rigidity, behaving like a viscous liquid (Carmo et al., 2017). According to de Souza et al. (2021), this process occurred due to a series of factors, from the initial execution of the dam construction, which was modified due to difficulties encountered and allowed for saturation zones, to the handling of the tailings, which facilitated the liquefaction process and subsequent rupture.

The discharged material consisted of water, solid particles of iron oxides and hydroxides, aluminum-bearing minerals, manganese oxides, and silica/quartz, along with trace concentrations of heavy metals such as lead, copper, and zinc (Samarco, 2017). Along its trajectory within the watershed, the material reached the Gualaxo do Norte and Carmo rivers, and finally the Doce River. In the Doce River, part of this material—already mixed with remobilized soil—was deposited in the Candonga Hydroelectric Reservoir, while the remainder continued as finer suspended loads toward the Atlantic Ocean (Interfederative committee, 2023). Thus, in addition to discharging a large volume of iron-ore tailings into river valleys, the Fundão dam failure generated a massive flow of mud and debris characterized by a high concentration of suspended sediment and elevated levels of metals and other contaminants (Bastos et al., 2017; Quaresma et al., 2020). These contaminants were associated both with the tailings released from the Fundão dam and with soils deposited along river channels and floodplains (Bastos et al., 2017; Hatje et al., 2017; Quaresma et al., 2021), which were remobilized during the passage of the mud and debris flow.

The large amount of substances—whether originating from the tailings or remobilized along the affected areas—drastically altered the landscape and biodiversity of the Doce River basin, devastating portions of Atlantic Forest and leaving extensive slopes and floodplains with exposed soil (Interfederative committee, 2018). Furthermore, coastal and marine areas adjacent to the watershed were also affected (Fig. 1). Visual interpretations of satellite images estimate that the tailings covered an area of up to 47,000 km^2^—mainly of lower-concentration plumes (IBAMA, 2017; ICMBio, 2017), spreading both southward (Rio de Janeiro state) and northward (to the Abrolhos Marine National Park, on the coast of Bahia state). This immense extent of the tailings resulted in alterations in beaches, sandbanks, mangroves, and the shallow continental shelf, and consequently impacting the biota of these ecosystems (Macêdo et al., 2024).

**Fig. 2:**
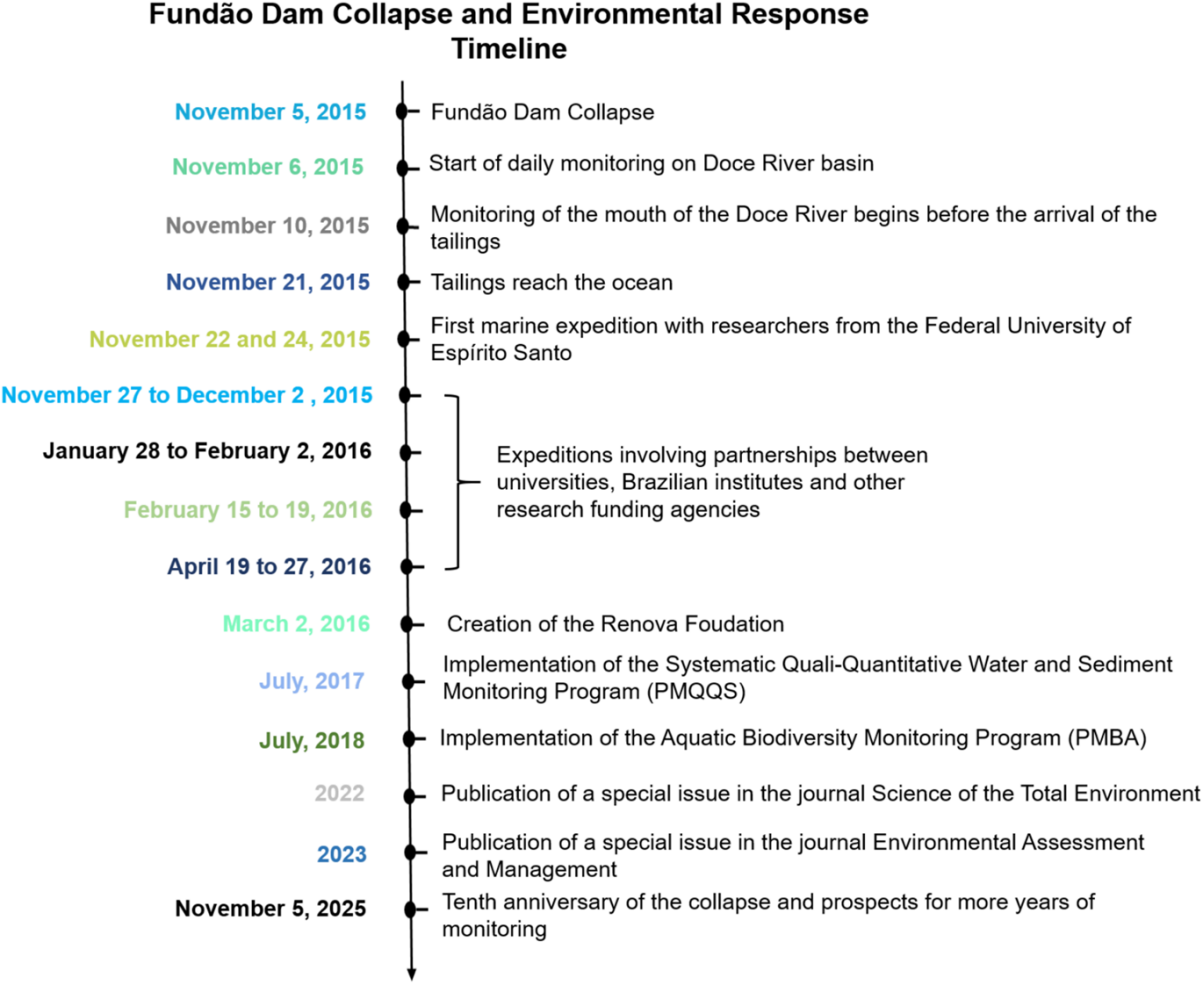
Timeline of events since the Fundão dam collapse

As a result, monitoring became necessary to evaluate the impacts caused by the tailings in these environments. Shortly after the collapse, researchers organized efforts to conduct sampling prior to the arrival of the tailings in downstream areas—given the scarcity of previous studies in the affected region—as well as immediately after the arrival of the mud flow, which revealed acute alterations and impacts (Fig. 2). In this sense, the Brazilian Geological Survey (CPRM, in Portuguese) and the National Water Agency (ANA, in Portuguese) organized daily collections starting on November 6th to track the movement of the tailings wave, also monitoring the quality of the water and sediments.

Samples were also collected in coastal and marine environments in the first days and months after the arrival of the tailings. The first collections in the Doce River mouth (characterized throughout the manuscript as the area between the municipality of Linhares and the South Atlantic Ocean) region were made by a group of researchers from the Federal University of Espírito Santo, who performed collections before the arrival of the tailings in that region (November 10, 12, 19 and 21, 2015), shortly after its arrival (November 22 and 23), and also 3 months later (February 3, 2016).

In the marine environment, the first expeditions were carried out independently by researchers from the Federal University of Espírito Santo between November 22 and 24, 2025. Furthermore, scientific expeditions were also carried out involving partnerships between universities, the Brazilian Institute of Environment and Renewable Natural Resources (IBAMA), the Chico Mendes Institute for Biodiversity Conservation (ICMBio), and other research funding agencies. The first expedition took place between November 27 and December 2, 2015, aboard the Navy ship Vital de Oliveira; the second expedition took place between January 28 and February 2, 2016, aboard the ICMBio ship Soloncy Moura; the third expedition took place between February 15 and February 19 aboard the Navy ship Antares; and the fourth between April 19 and 24, 2016, again at the Soloncy Moura ship. These expeditions aimed to assess the impacts of the mud’s arrival in the ocean and served as important baselines for historical comparisons.

At the same time, a governance framework was created to address the Fundão dam collapse, involving the responsible companies (SAMARCO, Vale S.A., BHP Billiton Brasil Ltda), the Federal Government of Brazil (IBAMA, ICMBio, ANA, DNPM, FUNAI), and the states of Espírito Santo (IEMA, Instituto de Defesa Agropecuária e Florestal do Espírito Santo—IDAF, AGERH) and Minas Gerais (IEF, IGAM, FEAM) (Lima et al., 2021). This governance framework established an agreement designed as a comprehensive instrument for implementing repairs and monitoring the damage caused by the dam collapse. To this end, the Renova Foundation (Fig. 2) was created, becoming the organization responsible for 42 environmental, social, and economic recovery programs for the parties involved.

Among these programs, two stand out for their focus on environmental issues and are primarily responsible for the ongoing monitoring efforts aimed at identifying long-term chronic impact patterns: the Systematic Qualitative and Quantitative Water and Sediment Monitoring Program, established in 2017, and the Aquatic Biodiversity Monitoring Program, established in 2018 (Fig. 2). These initiatives involve hundreds of researchers from various universities, national institutions, and also private companies, enabling the creation of a large information database and a deeper understanding of the impacts caused by the dam collapse.

Based on these research monitoring efforts, a series of studies were published addressing the alterations caused by the collapse in freshwater, coastal, and marine environments, employing approaches that considered both biotic and abiotic aspects. It is important to note that November 2025 marked 10 years since the collapse, with prospects for continued environmental monitoring for the next years. This reinforces the need for a critical evaluation of all studies conducted in the affected areas to establish the observed impacts and support decision-makers in identifying the main problems that still need to be addressed.

In this sense, the present study aims to conduct a review of articles that investigated alterations in the Doce River basin and adjacent coastal and marine areas as a result of the Fundão dam collapse. To this end, the studies were assessed according to their methodological approaches, spatial and temporal variations, environmental compartments analyzed and monitored topics of study. Additionally, the main findings were synthesized to highlight the alterations caused by the Fundão dam collapse in the Doce River basin and adjacent coastal and marine areas. Finally, recommendations are suggested for future integrative assessments between impacted environments.

## Methodology

**Fig. 3:**
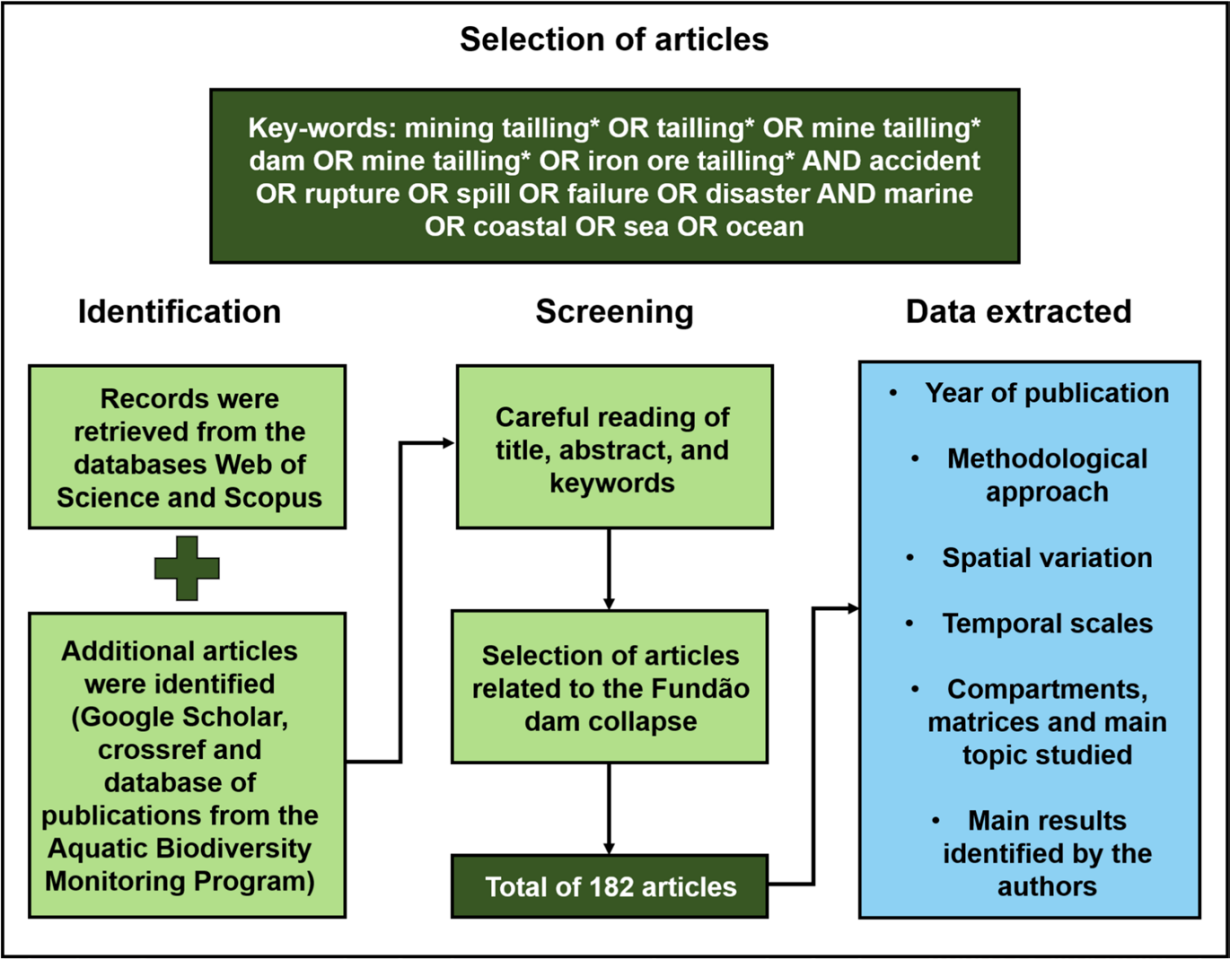
Flowchart of steps for obtaining the articles used in this study

This review was conducted between March 2023 and July 2025 using a bibliographic search protocol that helps identify articles on a given subject (Fig. 3). Initially, searches for articles were performed in the Scopus, Web of Science, and Google Scholar databases, using keywords combined with Boolean logic. In addition, other articles were retrieved through the database of publications from the Aquatic Biodiversity Monitoring Program (PMBA, in Portuguese) and from the reference lists of the selected articles.

For this research, articles between 2015 and 2025 were filtered, and the following keyword search sequence was applied: mining tailling* OR tailling* OR mine tailling* dam OR mine tailling* OR iron ore tailling* AND accident OR rupture OR spill OR failure OR disaster AND marine OR coastal OR sea OR ocean (Fig. 3). Subsequently, several steps were followed to retain only articles related to the Fundão dam collapse: (1) duplicate records across the databases were removed using RStudio software (Posit team, 2024); (2) dissertations, theses, technical reports, conference and symposium abstracts, reviews, and notes were excluded; and (3) a careful reading of the title, abstract, and methodology of each article was carried out, excluding those based solely on laboratory and/or ecotoxicological experiments. After this screening, a final set of articles remained from the databases, resulting in a total of 182 articles evaluated in this review.

After selection, the articles were carefully reviewed, and the following data were extracted from each of them: (1) year of publication; (2) methodological approach, categorized as observational/field sampling, database and/or document analysis, drone monitoring and/or ROVs, numerical modeling, and remote sensing; (3) spatial variation (classified as freshwater, terrestrial, coastal, and marine environments) and temporal scales (sporadic—without a fixed sampling trend; short-term—up to 2 years; medium-term—between 2 and 5 years; and long-term—more than 5 years); (4) compartments (abiotic and biotic), matrices (water, sediment, soil, and biota), and main topic studied (e.g., chemistry, ichthyology, phytoplankton and sedimentology; Supplementary material—Table 1); and (5) main results identified by the authors.

## Results and discussion

### Number of publications

**Fig. 4:**
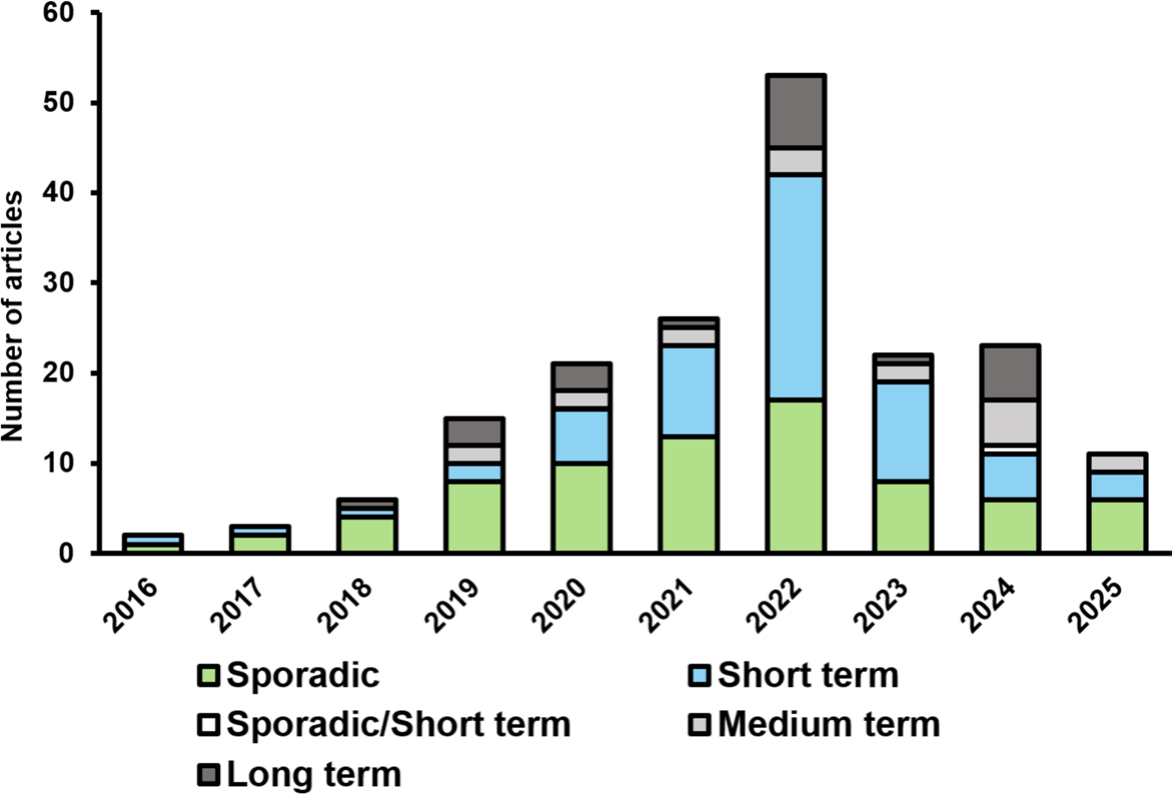
Number of articles and chronology of the temporal scales used in the studies reviewed. Sporadic studies are represented by light-green bars, short-term studies by black circles, medium-term studies by light-gray circles, and long-term studies by hatched circles. A single study employs more than one approach, conducting both sporadic and short-term sampling, and is represented by a white bar

As previously mentioned, the Fundão dam collapse occurred in November 2015, and from that point on, studies began to assess the environmental impacts in freshwater, coastal, and marine environments. The first pioneering studies on this topic were published as early as 2016 (Marta-Almeida et al., 2016; Segura et al., 2016). However, during the first 3 years, i.e., between 2016 and 2018, only a small number of publications on the subject were available (Fig. 4), mainly related to changes in land use and land cover caused by the dispersion of waste material and also water contamination After this period, a second phase (2019–2022) showed exponential growth in the number of publications, mostly related to two main factors: (1) the beginning of environmental monitoring in late 2018—mainly the Aquatic Biodiversity Monitoring Program and the Systematic Qualitative-Quantitative Water and Sediment Monitoring Program, which generated a large amount of abiotic and biotic data available to researchers across different fields; and (2) a special issue published by the journal Science of the Total Environment dedicated to the disaster.

Subsequently, articles have continued to be published regularly, with more than 50 articles between 2023 and mid-2025—including another special issue in the journal Integrated Environmental Assessment and Management—demonstrating the continued relevance of this subject. This considerable volume of publications underscores the substantial investment in scientific research. In addition, it reveals an ongoing commitment from the scientific community to monitor developments and assess potential impacts.

It is also interesting to note that most studies were coordinated at Brazilian universities/institutions (173 in total), with 28 of them having partnerships with researchers from 17 other countries. Additionally, there are also articles coordinated by international researchers, but who have partnerships with Brazilian institutions (e.g., Colman et al., 2019; Marta-Almeida et al., 2016; Richard et al., 2020). Finally, there were also articles conceived exclusively by researchers from other countries using existing open databases (Kananizadeh et al., 2024; Oehrig et al., 2024). This information demonstrates the global relevance of the Fundão dam collapse and how international partnerships can help in a better understanding of its impacts.

### Methodological approach

One of the key aspects to be evaluated in environmental research is the methodological approach of the studies, which directly influences the quality and reliability of the results obtained by the authors, depending on the rigor and adequacy of the methods employed (Zhang, 2007). In such research, methodological choices—from experimental design to sampling and data analysis techniques—are essential for ensuring that the conclusions presented are robust and can be used by decision-makers. Currently, environmental monitoring studies have largely relied on traditional methods, which often require time-consuming and costly sampling campaigns (Pan et al., 2025; Yang et al., 2022), but at the same time provide important insights into the assessed studied topics. In addition, innovative and cost-effective monitoring systems are increasingly being adopted, supporting the evaluation of environmental indicators more rapidly and across broader spatial and temporal scales (Forio & Goethals, 2020; Zhong et al., 2024).

**Fig. 5:**
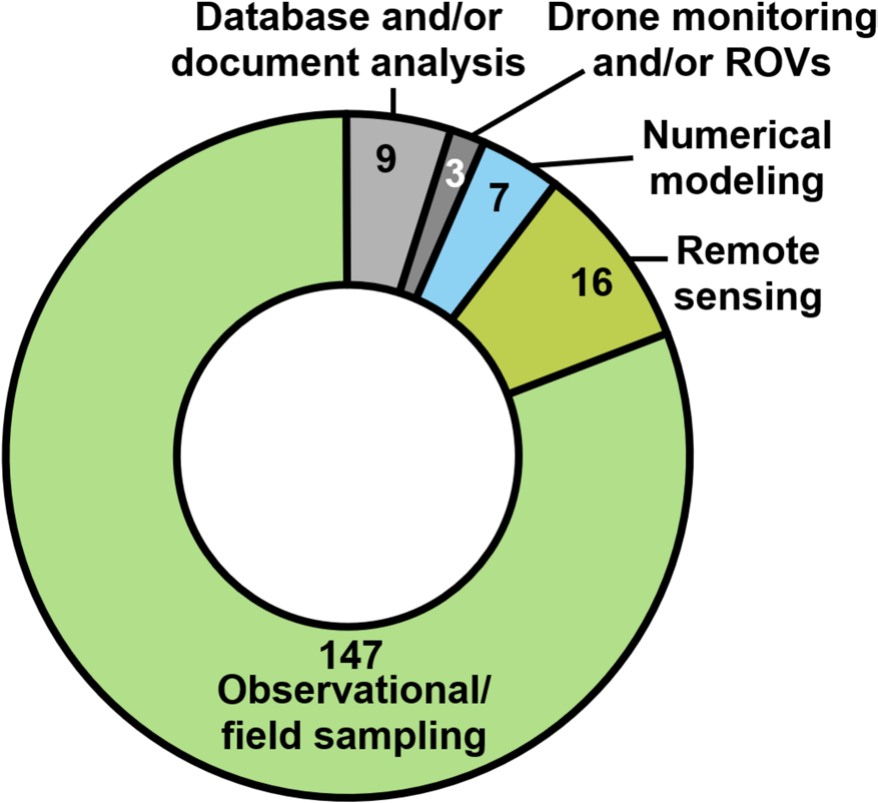
Distinct methodological approaches used by the 182 articles reviewed in the present study

Similar to the global panorama, the studies analyzed in this review were predominantly based on traditional environmental assessment methodologies, i.e., through field data collection and sample analysis (*n* = 147), representing 81% of the total articles (Fig. 5). There were also nine studies that analyzed databases or documents (*n* = 9), which similarly depend on observational and field data. This predominance is mainly due to the two large-scale environmental monitoring programs implemented in the region in 2017 and 2018—the Systematic Quali-quantitative Water and Sediment Monitoring and the Program Aquatic Biodiversity Monitoring Program—which have since provided regular field sampling, generating a substantial volume of data and information. This type of approach included the evaluation of a wide range of topics (see “Environmental compartments, matrices, and main topic of study” section), encompassing abiotic parameters, such as the concentration of organic and inorganic contaminants, physicochemical and granulometric parameters (Foesch et al., 2020; Hatje et al., 2017; Quaresma et al., 2021), as well as biological parameters, such as diversity, density, and biomass in field samples (Bonecker et al., 2022b; Coppo et al., 2023; Rocha et al., 2025).

On the other hand, some studies employed new systems and technologies to obtain their results, such as drone monitoring and/or ROVs (*n* = 3), numerical modeling (*n* = 7), and remote sensing (*n* = 16). The use of these tools enabled, for example, more accurate assessments of potential areas for mine tailing’s disposal (Aires et al., 2018); water physicochemical parameters (Miller et al., 2023; Miranda et al., 2023); dynamics of surface sediment concentrations (Aires et al., 2022) and tailings dispersion (Magris et al., 2019); as well as more efficient assessments of megafauna population dynamics (Barreto et al., 2021; Giacomo et al., 2021).

### Spatiotemporal efforts

Natural environments are not considered uniform and display high spatial and temporal variability (Chen et al., 2011; Cunillera-Montcusí et al., 2023), which can lead to distinct physicochemical and geological processes that, in turn, may result in changes in patterns and biodiversity. For this reason, it is essential to select the most appropriate temporal and spatial scales to achieve the study’s objectives and avoid the risk of misinterpretation. According to Nash (2014), the inadequate selection of analytical scales can lead to either overestimation or underestimation of the magnitude and significance of potential environmental effects, highlighting the need for a careful analysis of spatiotemporal factors. For example, studies conducted at shorter time scales and immediately after the collapse were able to demonstrate acute impacts of contaminants on the environment (e.g., Gomes et al., 2017; Loureiro Fernandes et al., 2020; Mourão et al., 2023). Conversely, sampling campaigns carried out over several years and with broader spatial coverage allow the identification of large-scale trends and patterns (e.g., Garcia et al., 2024; Kananizadeh et al., 2024).

In this context, the spatiotemporal scales of the selected articles were evaluated, considering both the environments in which the studies were conducted and the temporal scales chosen for analysis. To enable better standardization across studies, spatial variation was categorized by different environments, i.e., freshwater, terrestrial, coastal and marine, while temporal scales were based on Prado and Kleemann (2025) and classified as sporadic (without a fixed sampling trend), short-term (up to 2 years), medium-term (between 2 and 5 years), and long-term (more than 5 years).

Regarding temporal scales, it was observed that sporadic assessments—i.e., those based on sampling without a defined temporal pattern or consisting of only a single sampling event—were predominant, representing more than 40% of the studies reviewed, followed by short-term assessments (~ 36%) (Fig. 4). It is evident that until 2022 these approaches were more prominent, likely because many of the initial studies were designed to identify the acute impacts of the dam collapse and others relied on primary data from monitoring programs initiated in 2018, which did not allow sufficient time for medium-term and long-term approaches. Furthermore, it is worth highlighting that the initial sporadic and/or short-term studies served as an important baseline for the present study area, since few studies had been conducted prior to the failure.

From 2018 onwards, medium-term (*n* = 18) and long-term (*n* = 23) studies also emerged, made possible either by the availability of pre-collapse data and/or by the longer time elapsed since the disaster (e.g.Kananizadeh et al., 2024; Nunes et al., 2022; Vaneli et al., 2022). However, several authors indicate that there are still significant gaps in this type of approach, highlighting the need for more in-depth studies aimed at a broader temporal characterization of the environmental processes that drive abiotic and biotic changes (e.g.Coppo et al., 2023; Rocha et al., 2025; Santos et al., 2023). Currently, with 10 years since the collapse and 7 years of continuous monitoring, it has become possible to address these issues and better characterize the alterations and impacts across multiple levels and components of biodiversity in the lower Doce River and adjacent marine and coastal regions.

**Fig. 6:**
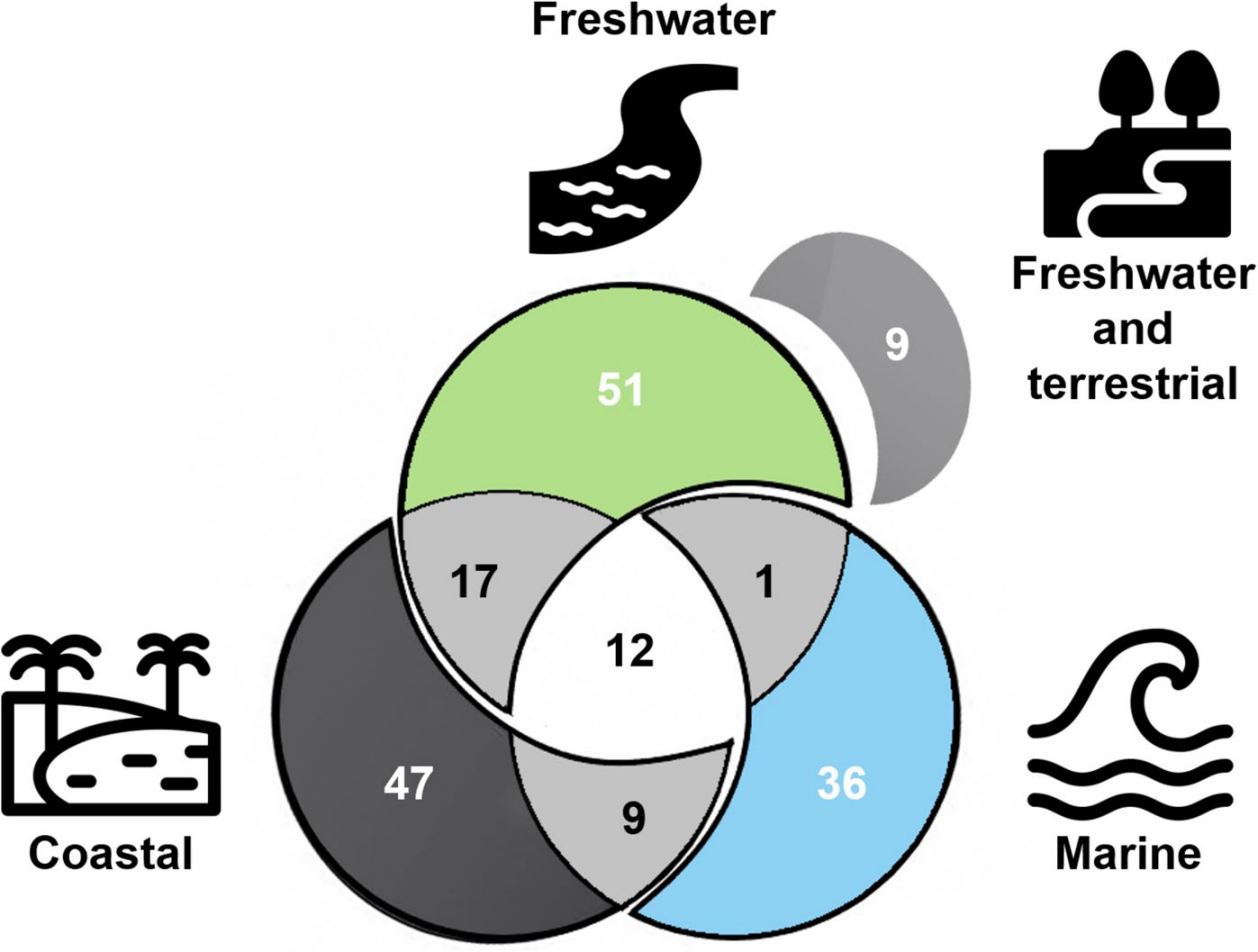
Spatial scope of the selected studies, separated into freshwater, terrestrial, coastal and marine environments, with the interactions between them

From a spatial perspective, it was possible to identify that most studies focus on a single environment (Fig. 6), with 51 articles conducted in freshwater systems (28%), 47 in coastal environments (26%), and 36 in marine settings (20%). This indicates that, despite these environments being intrinsically connected through abiotic and biotic linkages, there is a strong tendency to analyze them separately—similar to the divide often observed between aquatic and terrestrial ecosystems (Soininen et al., 2015). According to these authors, this occurs largely due to a historical issue reinforced by disciplinary boundaries between institutes and funding schemes, highlighting the need for new approaches that can more effectively integrate interconnected ecosystems.

Some studies, however, addressed more than one environment, particularly freshwater-coastal (*n* = 17), marine-coastal (*n* = 9), and freshwater-terrestrial (*n* = 9) interactions. Finally, only 12 studies simultaneously assessed all three environments (Fig. 5), with half relying on remote sensing or numerical modeling (e.g.Coimbra et al., 2019; Frachini et al., 2021; Miller et al., 2023), which facilitate a broader interpretation of data, and the other half based on field sampling (Cagnin et al., 2022; Costa et al., 2022; Evangelista et al., 2022; Sartori et al., 2023), demonstrating the feasibility of conducting more robust cross-environment analyses. Moreover, several authors of the reviewed studies emphasized the need for an increased number of integrative investigations encompassing multiple environments (Ferreira et al., 2020; Longhini et al., 2022; Suhadolnik et al., 2022), in order to achieve a more comprehensive understanding of the ecosystem as a whole.

### Environmental compartments, matrices, and main topic of study

According to Forio and Goethals (2020), aquatic ecosystems are composed of interactive elements that encompass abiotic characteristics—such as climatic, physical, chemical, and geological aspects—as well as biotic components. These authors further emphasize that these elements must be integrated and analyzed as a whole in order to achieve a holistic understanding of such ecosystems. In this context, the monitored compartments were assessed, namely abiotic or biotic, along with the matrices evaluated in each study (water, sediment, biota, and soil), and the main topic addressed. This approach aimed to identify potential interactions among the monitored components and to highlight existing knowledge gaps.

**Fig. 7:**
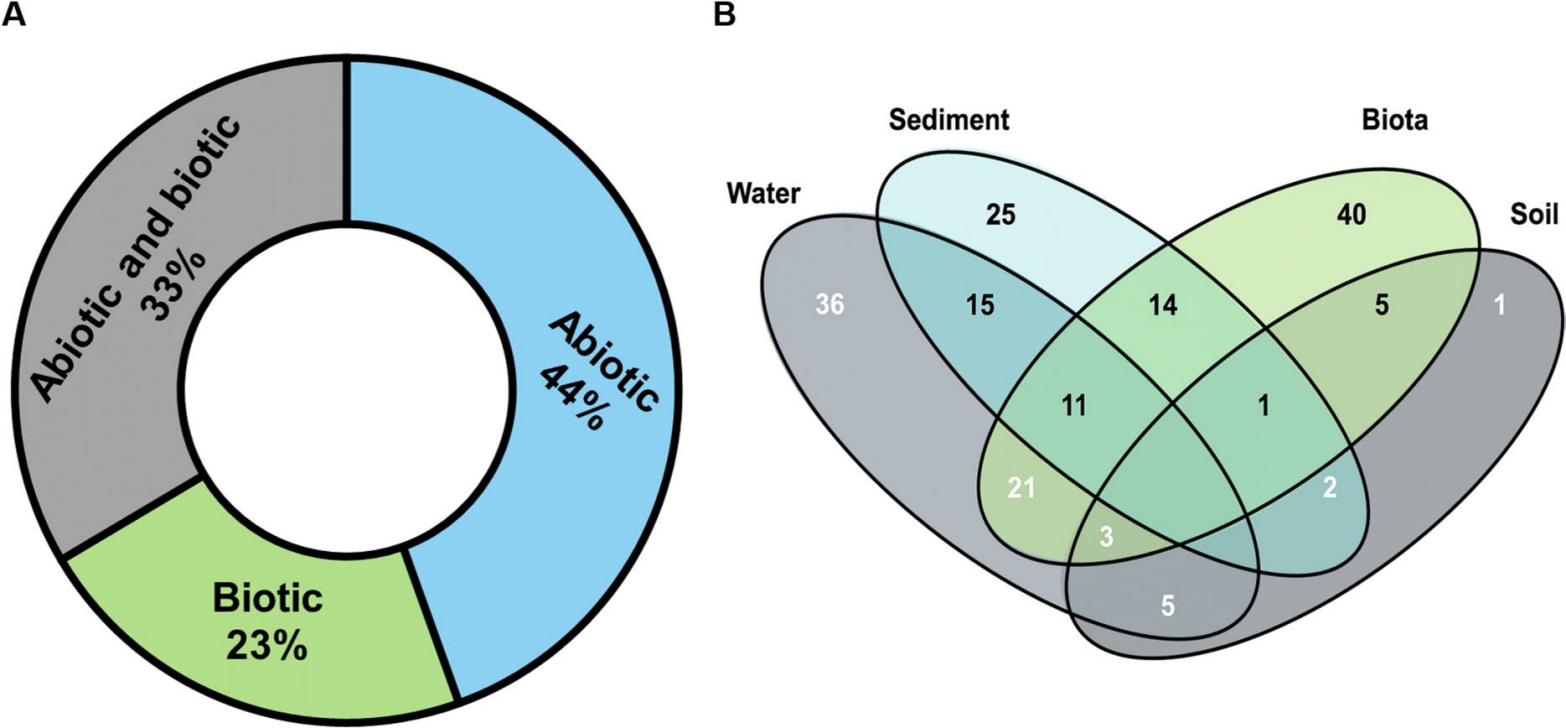
**A** Percentage of monitored compartments in the evaluated studies. **B** Different environmental matrices addressed by the studies. The number of articles performing analyses in each matrix (i.e., water, sediment, biota, and soil) is shown, with the intersection values representing the integration among them

The first aspect examined concerned the monitored compartments. It was observed that the abiotic domain received greater attention from researchers (Fig. 7a), largely due to the previously mentioned fact that the tailings carried high concentrations of contaminants throughout the Doce River basin and adjacent coastal and marine areas, where they accumulated in both the water column and sediments (Oehrig et al., 2024; Richard et al., 2020). A considerable number of studies (*n* = 6034%) also interconnect abiotic and biotic domains, investigating the relationships between physicochemical parameters and contaminant concentrations in water/sediment with: (1) bioaccumulation in organisms and coastal vegetation (D’Addazio et al., 2023; Gudin et al., 2024; Maraschi et al., 2022); and (2) ecological, population, and physiological parameters of aquatic communities (Barrilli et al., 2024; Bonecker et al., 2022a; Rocha et al., 2022).

There are also studies that focused on biological aspects, highlighting impacts across various organism groups and addressing different topics such as species identification (Nepomuceno et al., 2021), community structure (Almeida et al., 2023), frequency of occurrence (Bonecker et al., 2019), ecological indices (Fráguas et al., 2025), biomass (Hobbs et al., 2020), and contaminant bioaccumulation (Zebral et al., 2022).

Regarding the different environmental matrices, it was possible to identify that most studies performed analyses on a single matrix, with 40 articles evaluating biota, 36 water, 25 sediment, and only 1 soil, which together represent 56% of the studies (Fig. 7b). This is due to the fact that, traditionally, studies have focused on sampling isolated environmental compartments—largely because of researchers’ expertise in a specific field of knowledge—even though it is already known that there is strong interconnectivity among them (Escher et al., 2020).

On the other hand, there were also studies that sought better integration among matrices, performing analyses on two or three simultaneously (Costa et al., 2022; Fadul-Souza et al., 2022; Weber et al., 2020). These connections were mainly established by evaluating water and biota (*n* = 21), water and sediment (*n* = 15), biota and sediment (*n* = 14), as well as water, sediment, and biota together (*n* = 11) (Fig. 7b). Soil, in turn, had few connections with the other matrices, possibly because it is more directly related to the terrestrial environment.

**Fig. 8:**
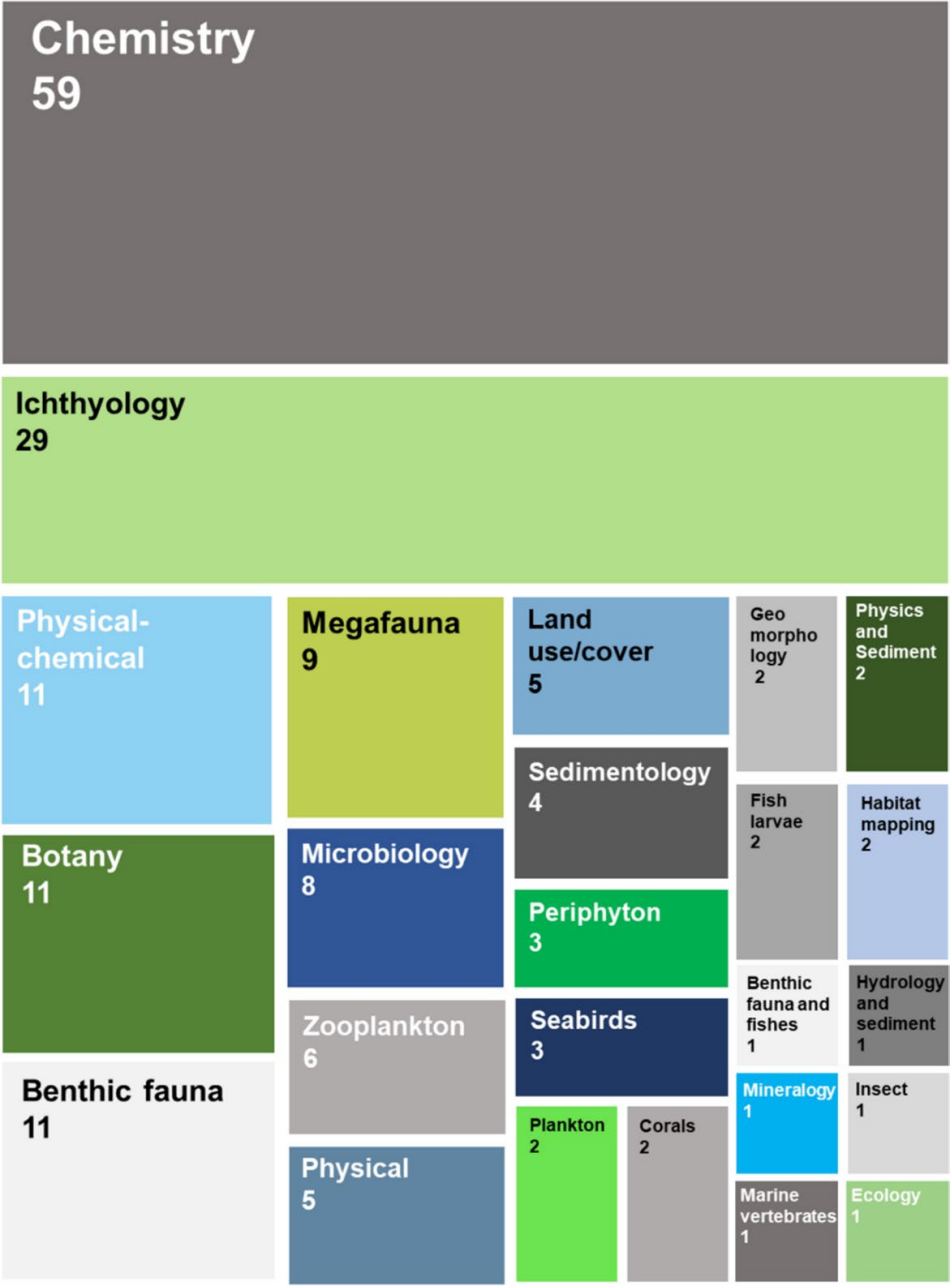
Number of articles on each topic. The size of the boxes represents the percentage of each topic in relation to the total number of articles

Figure 8 shows the main topics of study evaluated, making it possible to identify that chemistry was the primary focus (*n* = 59), representing approximately 32% of all studies. This number was influenced by the large input of chemical contaminants into freshwater, coastal, and marine environments, which encouraged researchers to investigate the impacts caused by them. The second most studied topic was ichthyofauna, with 29 published articles (~ 16%), addressing different aspects such as metal bioaccumulation (e.g.Bevitório et al., 2022; de Matos et al., 2022; Gabriel et al., 2020; Merçon et al., 2022), isotopic niches (Andrades et al., 2020; da Silva et al., 2025; Fráguas et al., 2025), ecological indices (Condini et al., 2022; De Biasi et al., 2023; Rodrigues et al., 2022), and even the introduction of exotic species in the affected area (Assis et al., 2024).

In addition, 23 other topics were identified, ranging from abiotic aspects such as physicochemical parameters (*n* = 11), physical oceanography (*n* = 5), land use and land cover (*n* = 5), sedimentology (*n* = 4), and geomorphology (*n* = 2), to biotic aspects, addressing a wide variety of groups, including microbiology (*n* = 4), periphytic communities (*n* = 3), phytoplankton (*n* = 2), zooplankton (*n* = 6), benthic communities (*n* = 11), botany (*n* = 11), seabirds (*n* = 3), and megafauna (*n* = 9) (Fig. 8). This breadth of topics demonstrates the variety of aspects that were considered when addressing the potential impacts caused by the collapse and highlights the complexity of integrative analyses that consider all environmental and ecological aspects.

### Key observations and trends

**Fig. 9:**
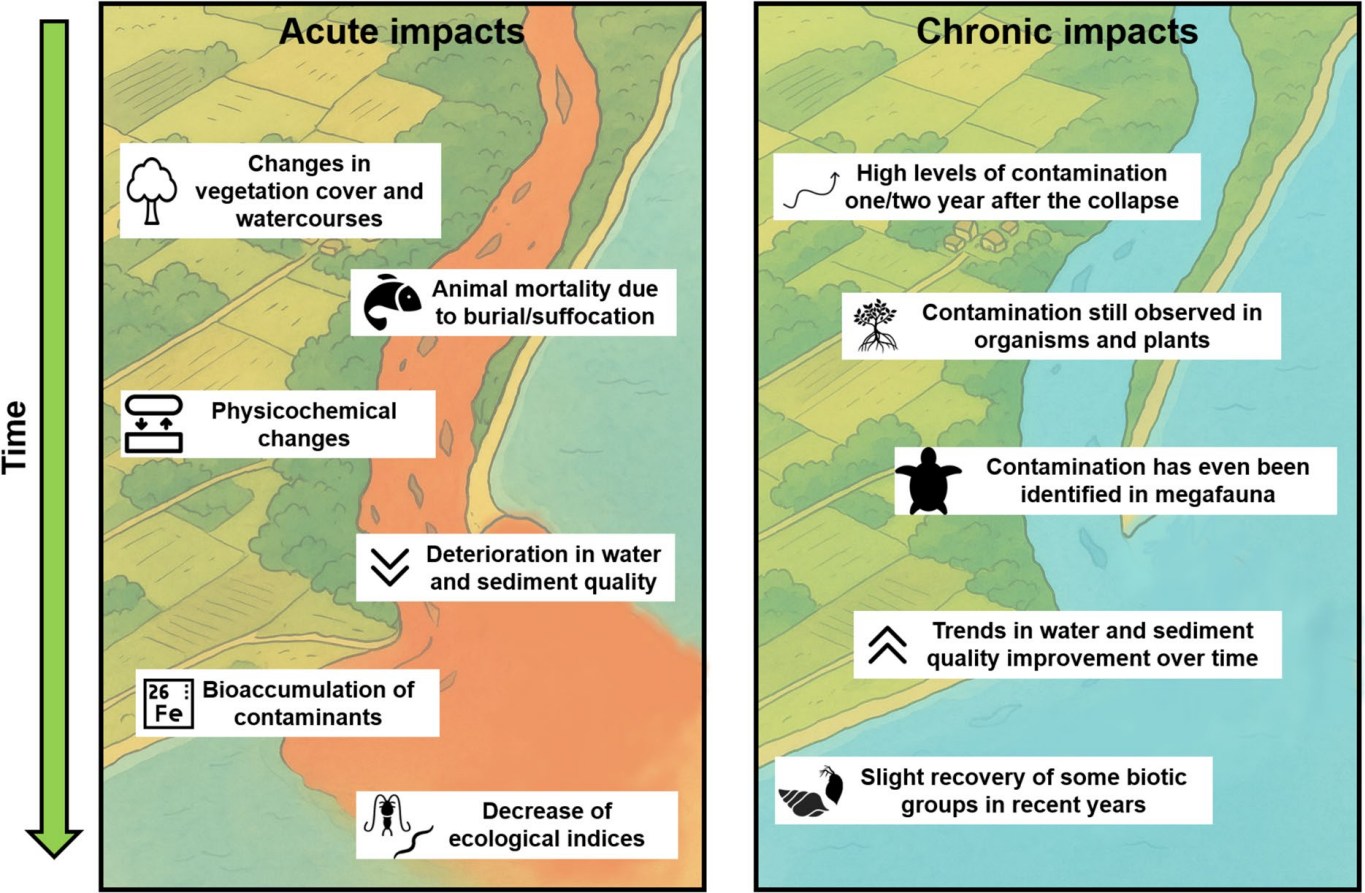
Key trends identified by the articles evaluated, showing acute and chronic impacts over time and what changes they caused in the environment and biota

After reviewing all the issues addressed in the previous sections—demonstrating that there are distinct approaches, whether methodological, spatio-temporal, environmental, or thematic—it became evident that greater integration among studies is needed. Such integration would enable a macro-scale perspective of the changes and impacts caused by the Fundão dam collapse. To this end, the present section sought to outline a timeline of the events and the corresponding studies, in order to highlight the main observations reported by researchers and to identify trends that could inform management and conservation plans (Fig. 9). The intention is not to present the results of each individual study, but rather to identify temporal patterns within each environment. A review conducted by Soares et al. (2025), which evaluated studies up to the year 2022, provides a clearer synthesis of the results from each article that performed water and sediment analyses.

The first point to consider in this integrative assessment is how changes in land use and land cover have been observed over time, primarily driven by the presence of mine tailings. Carmo et al. (2017), for example, showed that within only 12 h after the collapse, the tailings had reached 2,020 hectares, altering natural environments—such as native vegetation and agricultural areas, protected areas, watercourses, hydroelectric plants, and soils—as well as sociocultural aspects of the region, including residences, public, commercial, and religious buildings, and farms. Similarly, other studies identified major changes in vegetation cover and watercourses not only in areas close to the collapse but also in more distant regions (Aires et al., 2018; Coimbra et al., 2019; da Silva Junior et al., 2018). Furthermore, Eduvirgem et al. (2020), in a study with broader temporal coverage, indicated potential difficulties in the regeneration of arboreal vegetation, particularly along the margins of the Gualaxo do Norte River, with more areas of vegetation potholes identified in 2019 compared to 2013.

Another important topic evaluated was the dispersion of Fundão tailings in the Atlantic Ocean, which could reach key marine ecosystems as well as protected areas. In this context, studies sought to identify, through numerical modeling and satellite imagery, the possible locations reached by the tailings. These studies showed that the material may have dispersed over hundreds of kilometers, mainly southward (Marta-Almeida et al., 2016; Rudorff et al., 2018), but that it could also have been transported northward, especially when there is a shift in the wind pattern from northeast to south, potentially reaching the Abrolhos Bank in the state of Bahia (Coimbra et al., 2020; Magris et al., 2019).

As demonstrated in the previous sections, chemistry has drawn significant attention from researchers since the onset of investigations, due to the large number of contaminants that reached aquatic environments as a result of the mine tailings. Consequently, studies were conducted across different spatial scales in freshwater, coastal, and marine environments, as well as temporal scales, ranging from a few days before the collapse to 7 years after the event.

Early studies revealed a clear acute impact following the arrival of the tailings in the Doce River basin and adjacent marine areas. Substantial increases were observed in the concentrations of metals in water (both in dissolved and particulate forms), as well as in nutrients and polycyclic aromatic hydrocarbons, when compared with data collected days before the collapse, with values exceeding quality and regulatory guidelines (Costa et al., 2021; da Silva et al., 2024; Gomes et al., 2017; Mulholland et al., 2022; Sá et al., 2021). In addition, variations in the physicochemical parameters of water were also reported, with significant changes in suspended particulate matter and turbidity immediately after the collapse (Coimbra et al., 2019, 2020; Hatje et al., 2017; Kütter et al., 2023) (Fig. 9).

Following the event’s chronology, studies showed that concentrations of metals, nutrients (particularly nitrite and phosphate in the marine region), and polycyclic aromatic hydrocarbons reached even higher levels 1 year after the collapse (Costa et al., 2021; da Silva et al., 2024; Sá et al., 2021). Subsequently, water quality improved in the Doce River basin, with reductions in metal concentrations and physicochemical parameters (Garcia et al., 2024; Kütter et al., 2023; Oehrig et al., 2024), although occasional increases were identified during rainy periods in the region. Similarly, improvements were observed in coastal and marine areas impacted by the tailings in subsequent years—with the exception of the dry period in 2019, when more intense hydrodynamic forces, i.e., cold fronts with high waves that cause the resuspension of sediments, negatively affected water quality parameters (Longhini et al., 2022)—thus, presenting a possible environmental recovery over the years (Kananizadeh et al., 2024).

In the same way, acute contamination was also observed in the sediments of the Doce River as well as in the adjacent shallow continental shelf. For instance, Vergilio et al. (2021) reported metal enrichment in the Doce River sediments 15 days after the collapse, with higher concentrations found at sites closer to the tailings source area. Other studies characterized metal contamination in the Doce River mouth, identifying elevated concentrations of Al, Cr, Fe, Zn, and Ba 2 days after the arrival of the tailings in this region (Gomes et al., 2017), which increased even further after 7 days (Queiroz et al., 2018), when compared with pre-collapse data.

Studies also aimed to assess chronic sediment contamination across different environments. In the Doce River basin, Pauly et al. (2024) observed a clear decrease in metal concentrations 4 years after the dam failure, reaching values similar to those recorded prior to the disaster. Conversely, sediment samples collected in the upper Doce River 6 years after the collapse still indicated contamination, with concentrations exceeding Brazilian regulatory limits (Pires de Almeida et al., 2024). Furthermore, the mean concentrations found in this basin were higher than those recorded in marine and coastal environments, such as beaches and mangroves (Costa et al., 2022).

In the Doce River mouth, variations in metal concentrations in sediment were observed over time. Higher values were reported 1 year after the event (Sá et al., 2021), persisting at elevated levels for up to 2 years (Gabriel et al., 2021). This highlighted the continued role of this region as a source of tailings from Fundão to the coastal zone, being classified as the most polluted river mouth in the region. (Felizardo et al., 2021). After this period, concentrations decreased starting in 2018 and remained stable until 2020 (Gabriel et al., 2021). On the continental shelf, chronic metal contamination in sediments was also identified, with a significant increase in concentrations 1.5 years after the dam failure, along with notable alterations in metal fractionation (Aguiar et al., 2020; Quaresma et al., 2021). Three years later, elevated concentrations of certain metals (e.g., Zn, Cu, and Fe) were still detected, but subsequent declines were recorded in the following year (Longhini et al., 2022).

Biological communities were also assessed from the first days and months following the collapse. For instance, the microbial community in the Doce River basin exhibited short-term impacts, with an increase in soil-associated bacteria and alterations in metabolic profiles, as for example, genes related to the subsystems of microbial virulence, respiration, membrane transport, iron and nitrogen metabolism, and motility (Cordeiro et al., 2019). Furthermore, 25 days after the event, bioaccumulation of contaminants was already observed in fish from the Doce River basin, with concentrations of arsenic (As) and mercury (Hg) above Brazilian regulatory limits in some specimens (Mourão et al., 2023). In the mouth region, benthic macrofauna was negatively impacted, with declines in surface-dwelling taxa as well as reductions in both community diversity and evenness after the disturbance (Gomes et al., 2017). In the same region, shifts in fish niches were identified, indicating depletion of trophic diversity and basal resources across the community 6 months after the collapse (Andrades et al., 2020).

Similarly, biological communities in the marine region adjacent to the Doce River mouth experienced acute impacts. Zooplankton communities suffered an immediate loss of diversity, with a density peak dominated by opportunistic species (Fernandes et al., 2020). Fish larvae were also acutely affected, with some individuals presenting reddish sediment adhered to their bodies, damaged digestive tracts, and elevated metal concentrations (Bonecker et al., 2019).

In addition, short-term, medium-term, and long-term sampling campaigns were conducted with various groups of fauna and flora to assess the chronic impacts on aquatic biodiversity in the region. In freshwater environments, periphytic communities were altered due to metal concentrations, eutrophication, and light availability (caused by an increase in turbidity), with the Doce River mouth identified as the most impacted area (Zorzal-Almeida & Fernandes, 2021). Still within the basin, studies demonstrated the negative influence of physicochemical parameters (turbidity and conductivity) and metals (iron, zinc, and vanadium) on the species richness of zooplankton communities in both shallow and deep lakes, also affecting the functional diversity of this group of organisms (Santos et al., 2022, 2024).

Freshwater fish continued to show higher concentrations of As and Hg when compared to unaffected sites even 3 years after the arrival of the tailings (Ferreira et al., 2020). Evidence of reproductive disorders was also identified in specimens from the basin, which may negatively influence their population development (Merçon et al., 2021). Authors also demonstrated that the tailings affected the trophic metrics of the fish community in the basin, showing that areas in the upper course of the river were still undergoing significant fluctuations even 7 years after the rupture, whereas more distant regions displayed greater similarity to control areas (Fráguas et al., 2025).

In the Doce River mouth, metal concentrations were observed in wetland plant species, with bioaccumulation of Fe and Mn detected in roots, iron plaque, leaves, and shoots even 4 years after the collapse, and their bioremediation potential was discussed (Ferreira et al., 2022a, 2022b). According to these authors, these mechanisms enabled high rates of bioaccumulation of these elements, especially in the species *Typha domingensis*, concluding that it can be used as an important method for mitigating and restoring the impacts caused by the dam rupture. Similarly, metal contamination in fish was still present after 2 years, with concentrations above national and international limits for As, Cd, Cr, Cu, Mn, Pb, and Zn, indicating, according to the authors, a high risk to human health for consumers of these organisms (Gabriel et al., 2020).

For the composition of the benthic fauna, chronic impact was also observed on Doce River mouth, with iron and other metal contents being key factors in structuring the dominant meiofauna (Bernardino et al., 2019). The macrofaunal community was also affected, with lower richness and diversity values until 2019, and recovery of these community parameters in 2020 when compared to pre-collapse data (Coppo et al., 2023). In addition to metals, concentrations of rare earth elements—used as good proxies for tailings tracing (Cagnin et al., 2024)—were also observed in crabs from this region (Sales Junior et al., 2025), highlighting the contamination potential of these elements, even though they are found at low concentrations in the environment. Furthermore, even after more than 3 years of the collapse, decreases in the trophic diversity of the community were still detected during the rainy season, possibly caused by the remobilization of material present in the sediments (Andrades et al., 2021).

Biological assessments in the mangrove ecosystem were also conducted, monitoring forest structure, productivity, physiological aspects, and the population dynamics of crabs. These studies found metal bioaccumulation in the leaves of mangrove species at different sites (Tognella et al., 2022), leading to adjustments in their physiological responses, such as changes in chlorophyll synthesis and carbon assimilation (D’Addazio et al., 2023). For the mangrove crab *Ucides cordatus*, differences in population parameters were observed among the evaluated mangroves, due to distinct oceanographic conditions, geological aspects, and fishing pressure in each site (Lima et al., 2023). According to these authors, it was not possible to assess the acute impact of the dam collapse on this population because sampling was carried out 3 years after the event.

Regarding beach biota, studies were conducted both on restinga vegetation and on benthic fauna inhabiting these environments. In vegetation, possible negative effects on biochemical and physiological processes due to metals from the collapse were evaluated. For example, Oliveira et al. (2022) demonstrated that metal concentrations influenced fluorescence parameters in the species *Byrsonima sericea*. Alterations in photosynthetic parameters and reduced pollen grain viability were also identified in relation to the presence of metals (mainly Boron and Arsenic) in the herbaceous species *Canavalia rosea*, even 3.5 years after the collapse (Gudin et al., 2024). This same species, along with *Ipomoea imperati*, showed important responses to metal exposure, indicating potential adaptive mechanisms, such as adjustments in photosynthetic parameters (Lana-Costa et al., 2021). Furthermore, different studies also showed that other species present in the restinga region developed adaptive mechanisms, supporting their successful development even in affected areas (Lana-Costa et al., 2023; Lopes et al., 2024). The benthic fauna was also influenced by the collapse, with lower densities and species richness on dissipative beaches—a result contrary to global trends—possibly related to the higher concentrations of metals found in these areas (Brahim et al., 2024).

In the marine environment, chronic impacts were investigated in biological groups across different trophic levels, ranging from the phytoplankton community (primary producers) and zooplankton (primary consumers) to marine megafauna (top predators). Bonecker et al. (2022b) reported changes in the marine planktonic community 3 to 4 years after the collapse, with reduced phytoplankton abundance and diversity and lower zooplankton diversity associated with higher metal concentrations observed during the rainy season of 2019, due to increased contaminant inputs from the Doce River. Similarly, studies focusing on the zooplankton community showed that river discharge and the consequent influx of metals and inorganic particles were the main drivers of population dynamics up to 2020 (Rocha et al., 2022), with improvements in diversity, evenness, and the prevalence of bioindicator groups being identified from 2022 onward (Rocha et al., 2025).

The ichthyoplankton community showed similar patterns up to 2019, with lower abundance associated with increased discharge of the Doce River, in addition to many of the surface-collected eggs being non-viable (Bonecker et al., 2022a). Furthermore, Barrilli et al. (2024) demonstrated that the composition of fish larvae in the marine region adjacent to the Doce River was simplified compared to other areas, but has shown slight recovery in recent years.

The benthic macrofauna of the shallow continental shelf—particularly at sites closest to the Doce River mouth—was also chronically impacted, with the presence of tailings mud and elevated metal concentrations in the sediment being key structuring factors of community composition (Nascimento et al., 2022). According to these authors, there were drastic reductions in diversity and richness observed, with opportunistic species predominating even 4 years after the disaster. The spatiotemporal structuring of benthic fauna may also be linked to the bioaccumulation of contaminants by organisms, as observed for shrimp species in the marine region (Maraschi et al., 2022). Moreover, even sessile organisms were impacted by the dam collapse. Coral colonies located in the Abrolhos Bank (~ 250 km from the Doce River mouth) exhibited higher concentrations of metals in their skeletons after the disaster, with decreases in their growth rates also being observed (Cardoso et al., 2022; Evangelista et al., 2023).

Marine fish were also affected by the Fundão dam collapse, with higher levels of biological damage and metal bioaccumulation identified in areas closest to the Doce River mouth, demonstrating its influence (Bevitório et al., 2022). On the other hand, certain similarities were observed between ecological index patterns (diversity, richness, and evenness) and body mass indices in the marine region near the river mouth when compared to surrounding localities (Condini et al., 2022; Vilar et al., 2023), highlighting the need for further studies to obtain more conclusive standards.

Finally, marine megafauna was also studied in order to identify impacts resulting from the collapse, since many species of seabirds, turtles, and cetaceans are present in the marine and coastal region adjacent to the Rio Doce mouth (Giacomo et al., 2021). Zebral et al. (2022) reported significant spatio-temporal differences in metal contamination levels in birds, with higher values detected at the Doce River mouth and further south, particularly during the dry season of 2019. Moreover, birds collected in the Abrolhos Archipelago—but that forage in the area impacted by tailings—also showed elevated metal concentrations above the suggested threshold levels for some elements, even more than 5 years after the collapse, without, however, identifying substantial changes in foraging strategies (Bauer et al., 2024; Nunes et al., 2022). Two species of sea turtles (*Caretta caretta* and *Chelonia mydas*) were also affected even 3 years after the disaster, with higher concentrations of metals identified in specimens collected in areas near the Doce River mouth compared to control areas, potentially causing impacts on health and reproduction (Miguel et al., 2022b, 2023, 2022a). Bioaccumulation was also identified in franciscana dolphins (*Pontoporia blainvillei*), with temporal trends of increasing concentrations of some metallic elements (such as Hg and Zn) as well as organochlorine pesticides, which may result in toxic effects and influence the sustainability of the population (de Oliveira-Ferreira et al., 2022; Manhães et al., 2022).

## Future recommendations

**Fig. 10:**
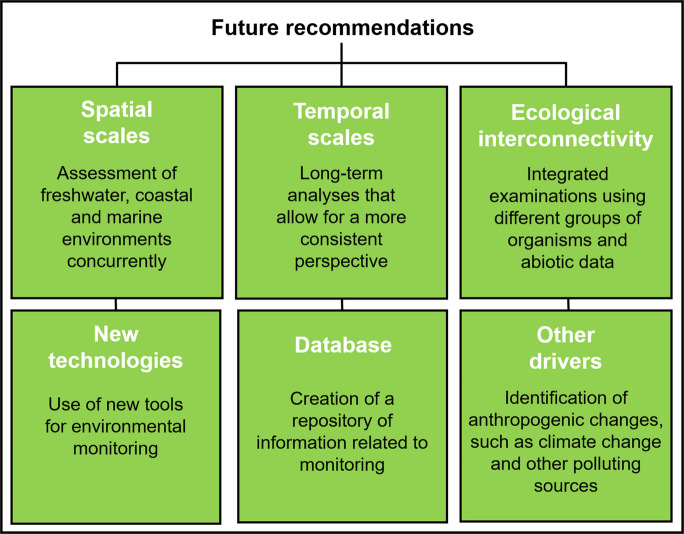
Recommendations provided for future studies, considering four main perspectives: spatial scales, temporal scales, ecological interconnectivity, and other impacts that may be observed in the affected area

After conducting this review, it was possible to identify some important aspects that should still be considered for consolidating the trends and observations presented in the previous section. To this end, recommendations are provided that should be considered by researchers for future studies (Fig. 10).

### Spatial scales

As demonstrated above, most of the studies analyzed performed sampling in a single environment, covering small to medium spatial scales. Therefore, studies that simultaneously assess freshwater, coastal, and marine environments are needed, considering a broader spatial scope that allows for a better connection among these ecosystems. This will enable the identification of processes and intra-environment relationships, providing a macro-scale perspective of environmental changes.

### Temporal scales

Among the articles analyzed, there was a predominance of sporadic and short-term studies, largely because the publications sought an immediate response to the dam collapse, due to the lack of prior data and the initial difficulty in establishing long-term monitoring. At present, however, consolidated and continuous monitoring programs have been in place for 7 years (Aquatic Biodiversity Monitoring Program and Systematic Qualitative-Quantitative Water and Sediment Monitoring Program), with possibilities of extending over the next 10 years. In this sense, it is suggested that future research should focus on long-term assessments, which would allow for the identification of clearer patterns and trends in the changes observed and potential recovery processes.

### Ecological interconnectivity

Largely due to researchers’ expertise, there is a strong tendency for studies to focus on a single topic. However, for a broader understanding of the environmental effects caused by the dam collapse, it is necessary to enhance ecological interconnectedness through the integrated assessment of different groups of organisms as well as abiotic data. In this sense, studies that investigate habitat integrity in all environments should be considered, serving as an important item to be considered in spatial planning, as has been developed, for example, in the European Marine Observation and Data Network (EMODnet).

### New technologies

Most of the studies were conducted through field sampling, which, as evidenced, entails high costs and significant time demands. In this context, we suggest the use of new technologies as effective tool for continuous and less-expensive monitoring. Currently, the concept of the Internet of Underwater Things (IoUT)—a network of intelligent, interconnected underwater devices such as sensors, autonomous underwater vehicles (AUVs), and robotic platforms, developed to monitor and explore aquatic environments—has been gaining increasing attention in several areas of aquatic research, assisting in the monitoring of physical, chemical, and biological parameters (Mohsan et al., 2023; Pieri et al., 2021). Therefore, a broader application of remote sensing, ROVs, AUVs, aquatic sensors, and drones is recommended to enable a more comprehensive understanding of the region’s environmental variables. Finally, using tools that allow for the creation of biogeochemical models can be useful in simulating the dynamic evolution of monitored ecosystems over the years.

### Database

Studies show that databases benefit science, enabling greater integration between isolated data and the consolidation of multidisciplinary approaches (Derrien et al., 2019; Luiza-Andrade et al., 2017). Therefore, the creation of an open-access repository of information related to the collected data should be another consideration for institutions and universities monitoring the impacts of the Fundão dam collapse on aquatic environments. This requires standard field measurements are necessary, including abiotic and biotic data across different environments, thus enabling comparisons between them.

### Other drivers

Beyond the environmental alterations caused by the Fundão dam collapse, other anthropogenic drivers can also be observed in the Doce River basin and adjacent coastal and marine areas, such as agriculture, urbanization, and mining activities (Vaneli et al., 2022). In addition, climate change may further affect the region by altering, for instance, temperature and precipitation patterns, which in turn influence river discharge and sediment transport to the coastal zone (Campos et al., 2024; May et al., 2020). Therefore, these impacts should also be considered in future studies, as they may represent additional causes for the observed changes in biotic and abiotic compartments.

## Conclusion

This study reviewed how the impacts caused by the Fundão dam collapse, which occurred 10 years ago, have been investigated, highlighting the methodologies employed, the different spatial and temporal scales, the monitored compartments, and the main topics analyzed. In addition, the main trends observed over time were presented, aiming to provide a macro-scale perspective that interconnects the various affected environments, thus forming a single meta-ecosystemic vision, which can, in turn, support the next steps to be taken by decision-makers.

Evaluating the publications over time was crucial for better understanding how interest and scientific production in a given field developed, providing insights into trends and relevance. This type of analysis enabled us to reconstruct the historical development of the topic by first establishing a clear timeline of events and highlighting the relevance of the earliest investigations. The initial studies were particularly crucial because they effectively served as a baseline, given the near absence of prior research in the study area. Subsequent studies, in turn, showed the progression of the possible environmental impacts. Furthermore, this assessment demonstrated the increase in production over the years, which is closely linked to the monitoring initiated in 2017/18, demonstrating its significant importance in terms of spatial and temporal issues.

In general, we conclude that freshwater, coastal, and marine environments have been well studied, revealing both acute and chronic impacts on abiotic and biotic compartments. When compared to other dam failures worldwide, the collapse of the Fundão tailings dam probably stands out as one of the most environmentally impactful events, largely due to the extensive dispersion of its tailings across freshwater, coastal, and marine ecosystems. On the other hand, it was also the one that maintained the longest monitoring period, which will likely continue for the next few years, allowing for a greater understanding and integration of the impacts caused. Through this extensive monitoring, it was possible to identify that aquatic environments suffered a significant acute impact, both on environmental and biological matrices. Studies generally showed that over time, chronic impacts tended to decrease, but there has not yet been a full recovery of the environment.

Moving forward, this review highlights the priority for future research to adopt integrated evaluations across these environments and leverage the now-available long-term data to better characterize chronic impacts and recovery trajectories. It is essential to ensure the continuity of the recovery measures implemented after the dam failure, including proper tailings management—such as dredging accumulated material, reconfiguring riverbanks to prevent slope instability, applying bioengineering techniques to restore and stabilize banks and riverbeds, and maintaining containment dikes and metal barriers—as well as the rehabilitation of springs, the restoration of Permanent Preservation Areas (APPs), and the revegetation of riparian zones. Furthermore, assessing the influence of other anthropogenic drivers alongside the collapse is necessary, as these additional pressures may exert significant influence on the region’s environmental and ecological aspects.

Finally, only through this macro-scale vision will it be possible to build the complete history of these ecosystems, which may, in turn, support management and conservation plans aimed primarily at environmental improvements, but also at social and economic benefits, given that these environments serve as sources of livelihood and culture for local populations.

## Supplementary Information

Below is the link to the electronic supplementary material.

ESM 1 (DOCX 13.6 KB)

## Data Availability

All data that supported the results of this study can be made available by the corresponding author upon reasonable request.
