# Supplementary material for: A decade after the Fundão Dam collapse: key findings from studies in the Doce River basin and adjacent coastal and marine environments
Source: Environ Monit Assess. 2026 May 7;198(5):560. doi: 10.1007/s10661-026-15356-4 (PMC13149703; doi:10.1007/s10661-026-15356-4)
Supplement: Supplementary file 1 — (DOCX 13.6 KB) [file 10661_2026_15356_MOESM1_ESM.docx]

**Supplementary material**

**Table 1:** List of topics covered by the evaluated articles.

| Benthic fauna | | Marine vertebrates |
| --- | --- | --- |
| Benthic fauna and ichthyology | Megafauna |  |
| Botany | Microbiology |  |
| Chemistry | Mineralogy |  |
| Corals | Periphyton |  |
| Ecology | Physical |  |
| Geomorphology | Physical-chemical |  |
| Habitat mapping | Physics/sedimentology |  |
| Hidrology/sedimentology | Plankton |  |
| Ichthyology | Seabirds |  |
| Ichthyoplankton | Sedimentology |  |
| Insect | Zooplankton |  |
| Land use and land cover |  |  |
